# Correction: Iannuzzi et al. Might Fibroblasts from Patients with Alzheimer’s Disease Reflect the Brain Pathology? A Focus on the Increased Phosphorylation of Amyloid Precursor Protein Tyr682 Residue. *Brain Sci.* **2021**, *11*, 103

**DOI:** 10.3390/brainsci13111588

**Published:** 2023-11-13

**Authors:** Filomena Iannuzzi, Vincenza Frisardi, Lucio Annunziato, Carmela Matrone

**Affiliations:** 1 Department of Biomedicine, University of Aarhus, Bartholins Allé, 8000 Aarhus, Denmark; filomena.iannuzzi@biomed.au.dk; 2 Geriatric and Neuro Rehabilitation Department, Clinical Center for Nutrition in the Elderly, AUSL-IRCCS Reggio Emilia, Giovanni Amendola Street, 42122 Reggio Emilia, Italy; vincenza.frisardi@ausl.re.it; 3 SDN Research Institute Diagnostics and Nuclear (IRCCS SDN), Gianturco, 80131 Naples, Italy; lannunzi@unina.it; 4 Division of Pharmacology, Department of Neuroscience, School of Medicine, University of Naples Federico II, 80131 Naples, Italy

## Error in Figure

In the original article [1], there was a mistake in “Figure 3”. During the assembly of Figure 3, Western blot panels labeled with APPpTyr, APP, and β-actin were mistakenly used for familiar patients with AD, AD, and healthy controls. This error also affected the optical density analysis of the APPpTyr levels reported in Figure 1 and Figure 3B, where the values corresponding to Figure 3 needed to be replaced. In contrast, APP and β-actins optical density analyses were performed on the correct panels that are now reported in Figure 3 and did not require changes in the corresponding Figure 3C–E. Nonetheless, these errors did not influence the overall significance of the results, which remain consistent with those reported and discussed in this article. The corrected *“*Figure 1 and Figure 3” appear below.

The authors apologize for any inconvenience caused and state that the scientific conclusions are unaffected. The original article has been updated.

## Figures and Tables

**Figure 1:**
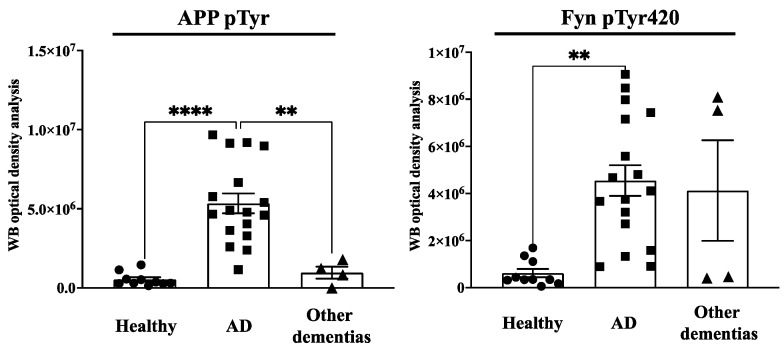
APP Tyr phosphorylation (APPpTyr) increases in fibroblasts from AD patients but not in patients with other forms of dementia or in healthy controls. The **left panel** reports the optical density analysis of APP pTyr bands, expressed as the mean optical density ratio between APP pTyr and APP basal levels from each sample in AD neurons (square) vs. healthy (circle) controls and other dementias (triangle). ** *p* < 0.005 and **** *p* < 0.0001, one-way ANOVA followed by Tukey’s test. The **right panel** reports the densitometric analysis of the bands expressed as the mean optical density (OD) ratio of Fyn pTyr420 relative to basal Fyn levels. ** *p* < 0.005, one-way ANOVA followed by Tukey’s test.

**Figure 3:**
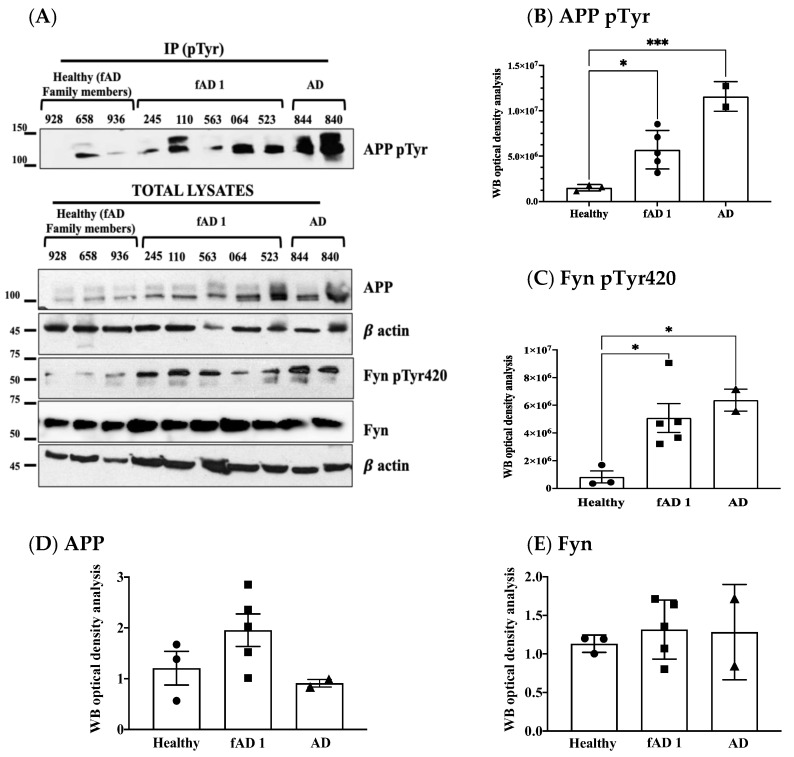
APP Tyr phosphorylation increases in fibroblasts from familial AD type 1 (fAD 1) and AD patients compared to healthy controls with a history of fAD 1. Panel (**A**) shows WB analyses of APP pTyr, Fyn pTyr_420_, APP and Fyn in healthy donors (circle) patients with a diagnosis of fAD 1 (square) and AD (triangle). Panels (**B**–**E**) report the densitometric analyses of the bands shown in panel (**A**). In particular, panel (**B**) reports the optical density analysis of APP pTyr bands expressed as the mean optical density ratio between APP pTyr and APP basal levels from each sample. * *p* < 0.05; *** *p* < 0.001. Panel (**C**) reports the ratio of FynpTyr_420_ relative to basal Fyn levels. * *p* < 0.05, one-way ANOVA followed by Tukey’s test. APP and Fyn expression levels, normalised on β-actin values, are reported in panels (**D**,**E**).
